# The Nail-Brush Question

**Published:** 1889-04

**Authors:** 


### Selections.

### The Nail-Brush Question.

Man is not instinctively clean. Cleanliness is the result of good training in childhood, and of common sense and self-respect in adult life. The doctrine that man is not by nature clean, nor even a seeker of what civilized people call comfort, may amply be proved by inspection of the members of a neglected family of children. Cleanliness and comfort represent acquired tastes, luxuries that have grown into necessities, items respected by social prejudice. Lastly, science has demonstrated that cleanliness and health are intimately allied.

The doctor has to pay great attention to cleanliness, for the laws of health and the good opinion of patients demand it. Dirty nails are abominable in every sense; hence the doctor constantly uses the nail-brush, both in the hospital ward and in the private sick-room. In the latter place there can be little risk in employing that article of toilet. Recently, however, the danger of the nail-brush in hospital work has been pointed out by more than one surgeon not without experience. The chief object of the nail-brush in the wards and out-patient rooms is not to make the surgeon's nails look beautifully clean. That is an important object, but the main aim of the use of the nail-brush is to remove any source of infection which may be carried from patient to patient. But the surgeon must perforce, under the ordinary arrangements which prevail in hospitals, make use of a brush which has repeatedly been employed by himself and his colleagues for removing from their nails a great many sources of infection. May he not then, be conveying, instead of removing, infection? In the course of a day's work in an out-patient room, a surgeon may repeatedly clean his nails with the same brush after his fingers have been soiled with pus, feces, and many other foul and morbid products. A colleague, about to perform an abdominal section, looks in on his out-patient colleague, and, perhaps, examines a case or two, taking care not to touch any morbid product. Having handled one or more men of toil, the surgeon rightly washes his hands to clean away that sweat or other allied kind of dirt, which is no doubt honest, but is likewise objectionable. He uses the nail-brush. It is evident that the chance of conveying septic matter will be increased, rather than diminished by its use, under the circumstances. A trace of dirt from healthy skin is less sceptic than the particles of filth already lying in the nail-brush, and fostered, perhaps, by the frequent immersion of the brush in warm water. The out-patient colleague can not have used water sufficiently hot to kill germs or decompose ptomaines. The destruction of infectious matter can not be insured by the weak solutions of antiseptics in vogue in out-patient practice. So the operation case may be put in jeopardy. More good may be done than harm by the nail-brush, no doubt, yet who can deny that, as above explained, it may do harm? There are several ways of overcoming the danger. A surgeon recently suggested that practitioners and operators who objected to nail-brushes should carry private brushes in their pockets. In private practice, however, the risk of using chance nail-brushes is but trivial. In hospitals it is otherwise. The surgeon might keep his own nail-brush in a locker, with other articles of toilet, if he please. Such lockers are seen in clubs and are not unknown in hospitals. In any case, scraping the nails with a penknife is far safer than using a strange nail-brush. By the well-known plan recommended by " D. H. G." (that soap should be forced under the nails and removed by means of the nails of the opposite hand), the skin beneath the nail is kept smoooth, and not roughened by a brush into traps to catch the dirt. No doubt both penknife and brush tend to make the nails rough, if not brittle. We have devoted to nail-brushes an amount of space in our columns which our readers will not deem excessive when they bear in mind the true importance of a subject which only at first sight appears trivial.—*British Medical Journal.*

THE *'Varsity* says: Oxford University is the largest in the world; it embraces twenty-one colleges and five halls. It has an annual income of $6,000,000.

